# The utility of the preoperative neutrophil-to-lymphocyte ratio in predicting severe cholecystitis: a retrospective cohort study

**DOI:** 10.1186/1471-2482-14-100

**Published:** 2014-11-27

**Authors:** Sang Kuon Lee, Sang Chul Lee, Jae Woo Park, Say-June Kim

**Affiliations:** Department of Surgery, Daejeon St. Mary’s Hospital, College of Medicine, the Catholic University of Korea, Daeheung-dong 520-2, Joong-gu, Daejeon, Republic of Korea

**Keywords:** Cholecystitis, Prognosis, Neutrophil-to-lymphocyte ratio, Length of hospital stay

## Abstract

**Background:**

To evaluate whether the neutrophil-to-lymphocyte ratio (NLR), as a prognostic indicator, in patients can differentiate between simple and severe cholecystitis.

**Methods:**

A database of 632 patients who underwent cholecystectomy due to cholecystitis during approximately a seven-year span in a single institution was evaluated. Severe cholecystitis was defined when the cholecystitis was complicated by secondary changes, including hemorrhage, gangrene, emphysema, and perforation. The NLR was calculated at admission as the absolute neutrophil count divided by the absolute lymphocyte count. We used receiver operating characteristic curve analysis to identify the optimal value for the NLR in relation to the severity of cholecystitis. Thereafter, the differences in clinical manifestations according to the NLR cut-off value were investigated.

**Results:**

Our study population comprised 503 patients with simple cholecystitis (79.6%) and 129 patients with severe cholecystitis (20.4%). The NLR of 3.0 could predict severe cholecystitis with 70.5% sensitivity and 70.0% specificity. A higher NLR (≥3.0) was significantly associated with older age (p =0.001), male gender (p =0.001), admission via the emergency department (p <0.001), longer operation time (p <0.001), higher incidence of postoperative complications (p =0.056), and prolonged length of hospital stay (LOS) (p <0.001). Multivariate analysis found that patient age ≥50 years (odds ratio [OR]: 2.312, 95% confidence interval [CI]: 1.472–3.630, p <0.001), preoperative NLR ≥3.0 (OR: 1.876, 95% CI: 1.246–2.825, p =0.003), and admission via the emergency department (OR: 1.764, 95% CI: 1.170–2.660, p =0.007) were independent factors associated with prolonged LOS.

**Conclusions:**

NLR ≥3.0 was significantly associated with severe cholecystitis and prolonged LOS in patients undergoing cholecystectomy. Therefore, preoperative NLR in patients undergoing cholecystits due to cholecystitis seemed to be a useful surrogate marker for severe cholecystitis.

## Background

Acute cholecystitis accounts for most of the hospital admissions related to gastrointestinal diseases [1]. In approximately 90% of patients, inflammation develops due to obstruction of the cystic duct by one or more gallstones [2]. Delayed management can lead to increased morbidity, due to progression to severe cholecystitis, such as gangrenous change, abscess formation, and gallbladder perforation. The prevalence of severe cholecystitis has been reported to be 22–30% in surgical series [3, 4]. Unfortunately, patients with severe cholecystitis are often challenging to accurately diagnose, both clinically and radiologically, since the clinical manifestations are unpredictable, and imaging studies are often equivocal [5]. However, marked contrasts in the morbidity and mortality rates have been observed beween patients with simple cholecystitis and severe cholecystitis [3, 6]. Therefore, prompt detection and proper management of patients at risk of severe cholecystitis are essential in preventing associated complications.

To predict the prognosis of inflammatory diseases and some malignancies, several inflammation-based scores have been suggested, including the Modified Glasgow Prognostic Score, neutrophil-to-lymphocyte ratio (NLR), platelet-to-lymphocyte ratio, and prognostic Nutritional Index [7, 8]. Of these, the NLR has received great interest, since it is simple to calculate, and involves no additional cost, as it uses results from a standard complete blood count test. The NLR is derived from the counts of circulating neutrophils and lymphocytes, both of which are major leukocyte subpopulations. The inflammation-triggered release of arachidonic acid metabolites and platelet-activating factors results in neutrophilia, and cortisol-induced stress results in relative lymphopenia, and thus, the NLR accurately represents the underlying inflammatory process [9]. Increasing evidence supports the utility of the NLR in predicting the prognosis of inflammatory and malignant diseases, although the application of the NLR to inflammatory gallbladder disease has not been reported. In the present study, we aimed to evaluate the utility of the NLR as a prognostic indicator in patients with cholecystitis, and to identify a relevant NLR value that discriminates between simple and severe cholecystitis.

## Methods

### Study design and data collection

We retrospectively reviewed prospectively collected data from patients who underwent cholecystectomies in Daejeon St. Mary’s Hospital, the Catholic University of Korea, between March 2007 and February 2014. Furthermore, we verified the current data, and obtained additional data, by including the radiology and pathology reports as a part of this study. This study was approved by the ethics committee, Daejeon St. Mary’s hospital, College of Medicine, the Catholic University of Korea (IRB code: DC13RISI0087).

During the study period, 1,023 cholecystectomies were performed either by the open or laparoscopic approach. To clearly identify gallbladder inflammation, we only selected 993 patients in whom the pathology reports indicated the presence of cholecystitis. Of these, we first excluded the patients (n =197) who initially presented with no specific symptom for cholecystitis. We then excluded patients (n =164) whose time-to-incision, the time interval between arrival to the hospital and the performance of surgical incision, was 120 h or longer. Finally, a toal of 632 patients were selected for inclusion in this study. Each patient’s NLR was calculated at admission as the absolute neutrophil count divided by the absolute lymphocyte count, using data from their standard complete blood count test.

We conducted receiver operating characteristic (ROC) curve analyses to determine the cut-off value for preoperative NLR that could discriminate between simple and severe cholecystitis. The most prominent point on the ROC curve was chosen as the cut-off value for the NLR. We then divided the patient population into two groups, according to the cut-off value of NLR, and attempted to detect differences in the clinical variables between these two groups. Thereafter, we performed univariate and multivariate analyses to investigate the effect of the NLR on the length of hospital stay (LOS).

### Terminology and definitions

Cholecystitis was defined by a histological finding of an inflammatory infiltrate on examination of the gallbladder wall. Severe cholecystitis was defined as a cholecystitis complicated by secondary changes, including hemorrhage, gangrene, emphysema, or perforation, and/or when the pathological examination indicated xanthogranulomatous cholecystitis; the representative forms of severe cholecystitis were gangrenous cholecystitis and gallbladder perforation. All other pathological findings were categorized as simple cholecystitis. Conversion was defined as the completion of any part of a procedure using an open technique except for minimal wound extension (≤10 mm) for specimen delivery. In addition, the incidence of an addition of ports during surgery was also counted. Operation time referred to the time interval between the initial skin incision and completion of wound closure, as documented by an anesthesiologist. With regard to intraoperative complications, bleeding referred to a loss of more than 200 mL during the operative procedure and gallbladder perforation referred to gross intraperitoneal bile contamination requiring irrigation. For postoperative complications, intra-abdominal hemorrhage referred to bleeding requiring transfusion, radiological, or surgical intervention; bile leak referred to persistent bile drainage from the drain site up to the 7th postoperative day or leakage requiring intervention; and voiding difficulty referred to the ongoing requirement for urinary catheterization up to the 7th postoperative day.

### Statistical analysis

Data were described as means ± standard deviations, or as medians and ranges. Continuous variables were compared using the independent *t*-test, while categorical variables were compared using the chi-squared test. Multiple regression analyses were performed using a proportional hazards model to identify factors independently associated with the LOS greater than the 80th percentile after cholecystectomy, and to estimate corresponding odds ratio (OR) in 95% confidence intervals (CI). Statistical analysis was performed using SPSS version 15.0 (SPSS Inc., Chicago, IL). Statistical significance was accepted for *P*-values less than 0.05.

## Results

### Determining the cut-off value of the NLR and comparison of preoperative variables

A total of 632 patients who underwent cholecystectomy owing to symptomatic cholecystitis during the study period were included in this study. The median age was 55.2 (13 – 91) years, and the patients comprised 335 women (53.0%) and 297 men (47.0%). Of these, 511 patients (80.9%) exhibited calculous cholecystitis and 121 patients (19.1%) exhibited acalculous cholecystitis. Pathologic examination confirmed the presence of simple cholecystitis in 503 patients (79.6%) and severe cholecystitis in 129 patients (20.4%). Regarding the admission route, 283 patients (44.3%) were admitted via the emergency department (ED) and 352 (55.7%) were admitted via the outpatient clinic.

**Figure 1:**
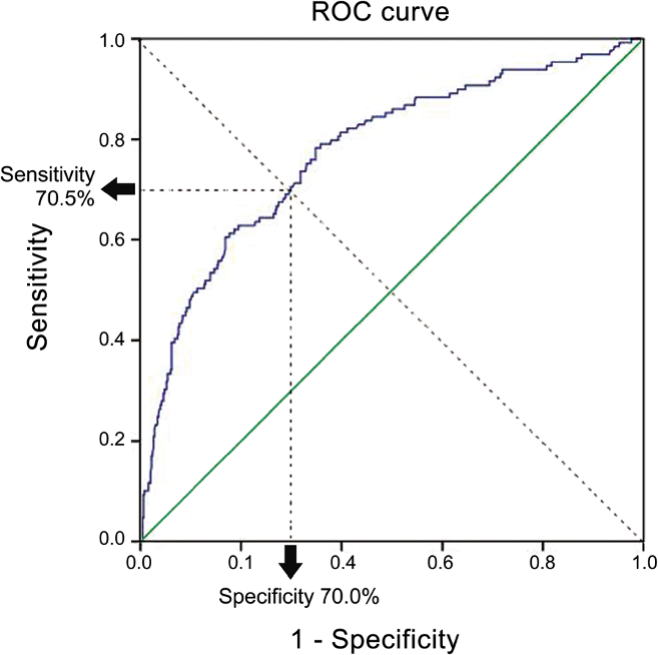
**Receiver operating characteristic (ROC) curve for simple and severe cholecystitis.** The area under ROC curve: 0.775. 95% CI 0.727 – 0.822; p-value <0.001.

An ROC curve was established to determine the cut-off value for preoperative NLR that could discriminate between simple cholecystitis and severe cholecystitis. The ROC area under the curve was 0.775. With an NLR value of 3.090, the sensitivity and specificity were 70.5% and 70.0%, respectively (Figure 1). Therefore, we considered 3.0 as the cutoff value, and divided the patient population into two groups: those with preoperative NLR values below 3.0 (n =376) and those with values equal to or greater than 3.0 (n =256).

When comparing preoperative variables, the two groups showed differences in age, sex, severity of cholecystitis, serum leukocyte count, presenting symptoms, and admission route; the higher NLR (NLR ≥3) group included more patients who had advanced age (p =0.001), were male (p =0.001), had severe cholecystitis (p <0.001), had higher leukocyte count (p <0.001), and had initially visited the ED (p <0.001) (Table 1). Both groups did not show any differences in other variables, such as the body mass index and gallbladder contents.

**Table 1:** **Demographic and preoperative characteristics of patients who underwent cholecystectomy due to cholecystits grouped by NLR**

	Total patients	NLR <3	NLR ≥3	p-value
	(n =632)	(n =376)	(n =256)	
Age, years	55.18 (13 – 91)	53.48 (17 – 91)	57.68 (13 – 89)	0.001
Sex				0.001
Men	297 (47.0)	156 (41.5)	141 (55.1)	
Women	335 (53.0)	220 (58.5)	115 (44.9)	
Body-mass index	24.51 ± 3.81	24.59 ± 3.86	24.35 ± 3.72	0.445
Previous abdominal surgery (%)	140 (22.2)	89 (23.7)	51 (19.9)	0.284
Severity of cholecystitis				< 0.001
Simple cholecystitis	503 (79.6)	342 (91.0)	161 (62.9)	
Severe cholecystitis*	129 (20.4)	34 (9.0)	95 (37.1)	
Content in the gallbladder				0.122
No	121 (19.1)	64 (17.0)	57 (22.3)	
Stone(s)	511 (80.9)	312 (83.0)	199 (77.7)	
Serum leukocyte count	7700	6200	11300	< 0.001
	(1500 – 39300)	(2700 – 16500)	(1500 – 39300)	
Presence of symptom				0.038
Abdominal pain	586 (92.7)	342 (91.0)	244 (95.3)	
Indigestion (discomfort)	25 (4.0)	19 (5.1)	6 (2.3)	
Fever/Chill	7 (1.1)	3 (0.8)	4 (1.6)	
Others	14 (2.2)	12 (3.2)	2 (0.8)	
Admission route				< 0.001
ED	280 (44.3)	120 (31.9)	160 (62.5)	
Outpatient clinic	352 (55.7)	256 (68.1)	96 (37.5)	
Time to incision				0.497
Mean ± SD	35.1 ± 27.0	34.5 ± 26.9	36.0 ± 27.2	
Median (range)	23.0 (1 – 120)	22.0 (1 – 120)	24.0 (2 – 119)	

*Abbreviations:* *ED* Emergency department, *NLR* neutrophil-to-lymphocyte ratio, *SD* standard deviation.

*Severe cholecystitis included emphysematous cholecystitis, gangrenous cholecystitis, xanthogranulomatous cholecystitis, and perforated cholecystitis.

### Comparison of operative and postoperative variables

Next, surgical outcomes were compared between the groups (Table 2). Cholecystectomies were performed mainly laparoscopically, using between one and four ports. The two groups showed no difference in the operative approach and conversion rates. However, the higher NLR group had a longer operation time (median values; 77.5 min vs. 65.0 min, p <0.001) and longer LOS (median values; 5.0 days vs. 3.0 days, p <0.001). In addition, Jackson-Pratt drains were more commonly placed in the higher NLR group (48.4% vs. 35.9%, p =0.002). The higher NLR group also showed higher incidence of postoperative complications, but it was not significant (4.3% vs. 8.2%, p =0.056).

**Table 2:** **Intra-operative and postoperative characteristics of patients who underwent cholecystectomy due to cholecystits grouped by NLR**

	Total	NLR <3	NLR ≥3	p-value
	(n = 632)	(n =376)	(n =256)	
Operative procedure				0.833
Open cholecystectomy	6 (0.9)	1 (0.3)	5 (2.0)	
Laparoscopic cholecystectomy				
- 4 port procedure	204 (32.3)	120 (31.9)	84 (32.8)	
- 3 port procedure	66 (10.4)	43 (11.4)	23 (9.0)	
- 2 port procedure	307 (48.6)	174 (46.3)	133 (52.0)	
- single port procedure	49 (7.8)	38 (10.1)	11 (4.3)	
Mean operative time (min)				< 0.001
Mean ± SD	76.5 ± 32.7	70.3 ± 25.4	85.6 ± 39.5	
Median (range)	70.0 (30 – 370)	65.0 (30 – 175)	77.5 (30 – 370)	
Insertion of a drain				0.002
No	373 (59.0)	241 (64.1)	132 (51.6)	
Yes	259 (41.0)	135 (35.9)	124 (48.4)	
Open conversion	4 (0.6)	2 (0.5)	2 (0.8)	0.485
Addition of one or more port(s)	5 (0.8)	4 (1.1)	1 (0.4)	
Frequency of total analgesics				0.385
Mean ± SD	3.3 ± 3.9	3.2 ± 3.7	3.5 ± 4.3	
Median (range)	2.0 (0 – 22)	2.0 (0 – 20)	2.0 (0 – 22)	
Postoperative hospital stay (days)				< 0.001
Mean ± SD	4.4 ± 2.8	3.8 ± 2.1	5.2 ± 3.4	
Median (range)	4.0 (1 – 24)	3.0 (1 – 23)	5.0 (1 – 24)	
Postoperative complications	37 (5.9)	16 (4.3)	21 (8.2)	0.056
Voiding difficulty	8	4	4	
Pleural effusion	8	4	4	
Wound infection	5	2	3	
Pneumonia	5	2	3	
Bleeding	4	2	2	
Bile leakage	3	1	2	
Pneumothorax	1	1	0	
Pancreatitis	1	0	1	
Delayed gastric emptying	2	0	2	

*Abbreviations:* *NLR* neutrophil-to-lymphocyte ratio, *SD* standard deviation.

### Analysis of factors affecting the LOS

Overall, the median LOS was 4.0 days (range, 1–24 days), and the 80th percentile of LOS was 6 days. We then divided patients as either control (LOS <6 days) or those with prolonged LOS (≥6 days) (Table 3). Univariate and multivariate analyses were conducted to identify the factors associated with prolonged LOS. On univariate analysis, patient age (p <0.001), male gender (p =0.036), preoperative leukocyte count (p =0.001), preoperative NLR (p <0.001), and admission via the ED (p <0.001) were all associated with prolonged LOS. A subsequent multivariate analysis identified that patient age ≥50 years (OR 2.321, 95%CI 1.47–3.630, p <0.001), preoperative NLR ≥3.0 (OR 1.876, 95% CI 1.246–2.825, p =0.003), and admission via the ED (OR 1.764, 95% CI 1.170–2.660, p =0.007) were independent factors associated with prolonged LOS.

**Table 3:** **Odds ratio for increased length of hospital stay (≥6 days; 80percentile) associated with clinical variables in patients undergoing cholecystectomy due to cholecystitis**

	Univariate analysis			Multivariate analysis		
	Odds ratio	95% C.I.	p-value	Odds ratio	95% C.I.	p-value
Age			< 0.001			< 0.001
< 50 years (standard)						
≥ 50 years	2.553	1.642 – 3.970		2.312	1.472 – 3.630	
Sex			0.036			
Woman (standard)						
Man	1.508	1.036 – 2.195				
Body-mass index			1.000			
< 25 (standard)						
≥ 25	0.988	0.677 – 1.440				
History of prior laparotomy			0.491			
No (standard)						
Yes	0.829	0.521 – 1.318				
Preoperative leukocyte count (/mm^3^)			0.001			
< 10,000 (standard)						
≥ 10,000	1.928	1.307 – 2.844				
Preoperative NLR			< 0.001			0.003
< 3.0 (standard)						
≥ 3.0	2.482	1.696 – 3.633		1.876	1.246 – 2.825	
Admission via ED			< 0.001			0.007
No (standard)						
Yes	2.172	1.485 – 3.178		1.764	1.170 – 2.660	
Approach of operative method^*^			0.613			
Conventional surgery						
Reduced port surgery	0.892	0.602 – 1.321				

*Abbreviations:* *ED* emergency department, *NLR* neutrophil-to-lymphocyte ratio.

*Conventional surgery included open surgery, 3-port and 4-port cholecystectomies, and reduced port surgery includes 2-port and single-port laparoscopic cholecystectomies.

## Discussion

Since severe cholecystitis is associated with more adverse clinical features than simple cholecystitis, prompt detection of the severe cholecystitis and surgical intervention before its further advancement is essential to avoid complications related to advanced histology. Patients with severe cholecystitis are at an increased risk of damage to the main biliary ducts, ligation of aberrant hepatic ducts, and injury to the right hepatic artery during surgery [10]. Thus far, the detection of severe cholecystitis has typically relied on imaging studies, such as abdominal ultrasound and computerized tomography (CT) scanning. However, these studies occasionally fail to detect severe cholecystitis [2, 5]. In the present study, we demonstrated that a preoperative NLR of 3.0 has the potential to differentiate between simple and severe cholecystitis. Therefore, the NLR calculation could be used to determine surgical priority by improving diagnostic accuracy when the CT findings are ambiguous, and by predicting a patient’s risk of progressing from simple to severe cholecystitis.

Patients with advanced inflammatory or malignant diseases usually present with elevated NLR as a manifestation of the systemic inflammatory response. Many investigators have attempted to identify the association between the NLR and its underlying molecular basis, and have found that there is an elevation in the levels of pro-inflammatory cytokines (e.g., IL-1ra, IL-6, IL-7, IL-8, IL-12) in the plasma of patients with elevated NLR [11–13]. These inflammatory cytokines are expected to perpetuate a tissue microenvironment favoring aggressive inflammation or tumoral behavior. Furthermore, high peritumoral infiltration of macrophages was observed in cancer patients with elevated NLR [12]. Therefore, elevated NLR appears to be an accurate indicator of up-regulation of the innate immune response.

The representative forms of severe cholecystitis are gangrenous cholecystitis and gallbladder perforation. Gangrenous cholecystitis occurs in up to 30% of patients with cholecystitis [5], wherein inflammation causes interruption of blood flow to the gallbladder, resulting in gangrenous change. The mortality rate was reported to be up to 22%, and it is directly related to other severe complications, such as gallbladder perforation, abscess formation, and peritonitis [14]. Gallbladder perforation is the eventual result of severe cholecystitis, where inflammation can either be localized or spread throughout the whole abdominal cavity via the perforated gallbladder [15]. In both forms, inflammation is expected to result in elevated NLR, which validates our results.

This study shows the usefulness of preoperative NLR in predicting prognosis and therefore, in determining operative priority in patients with cholecystitis. Patients with acute severe cholecystitis have higher incidences of postoperative complications and a prolonged LOS [16–19]. In this study, high NLR was found to be a predictor of severe cholecystitis as well as an independent risk factor for prolonged LOS. Early cholecystectomy was shown to decrease LOS in patients with acute severe cholecystitis [20]. Therefore, prioritizing patients with high NLR for operation would reduce postoperative morbidity and LOS. Similarly, operation time was longer in the high NLR group than in the low NLR group. However, the incidences of open conversion or the addition of one or more port(s) were similar in the two groups.

To our knowledge, this study is the first to investigate the relationship between the NLR and cholecystitis. According to the disease entities or their severity, a range of NLR cut-off values have been proposed, usually from 3 to 8 [21]. Of these, a threshold of >5.0 has been most frequently proposed [22–26], while recent reports have recommended a value of 3.0 [27–31]. We determined the cut-off value of severe cholecystitis as 3.0 based on our ROC curve analysis; the NLR value of 3.0 had an acceptable reliability in the analysis (70.5% sensitivity and a specificity of 70.0%). Therefore, we believe that a NLR cut-off value of 3.0 is suitable, and consistent with previous studies [27–31]. However, further study is needed to validate our cut-off value, and to more precisely identify an optimal NLR with the greatest prognostic power in cholecystitis.

The limitations of this study are those common to all database research. Since it involved a retrospective review of prospectively collective data, the results should be confirmed by prospective trials. Moreover, when we divided our patient population according to the NLR cut-off value, patient distribution was not well-balanced; our higher NLR patients included more patients with older age and men. Therefore, age and sex may act as confounding factors, which may affect the conclusion that an NLR of 3.0 is independently related with the severity of cholecystitis. In addition, the incidence of acalculous cholecystitis herein was 19.1% which did not fall within the general range of 2% to 15% as noted by others [32, 33]. Acute acalculous cholecystitis has been generally shown poor prognosis than acute calculous cholecystitis; the median NLRs of calculous cholecystitis and acalculous cholecystitis were 2.67 and 2.18, respectively (P =0.004). Therefore, in understanding our results, the differences in the composition of patient population should be taken into consideration.

## Conclusions

Routine preoperative NLR calculation in patients with cholecystitis did not only provide a simple means of identifying patients with severe cholecystitis, but also served as a surrogate marker for predicting prolonged LOS. We found that the patients with cholecystitis could be divided into low risk for severe cholecystitis (NLR <3.0) and high risk for severe cholecystitis (NLR ≥3.0) according to the NLR value at admission. Such approach of determining the operative priority based on the NLR value is expected to induce favorable surgical outcome by satisfying the "sickest first" principle and enabling expectant perioperative management

## Abbreviations

CI: Confidential interval
LOS: Length of hospital stay
NLR: Neutrophil-to-lymphocyte ratio
OR: Odds ratio
ROC: Receiver operating characteristic.
